# Cocoa and Heart Health: A Historical Review of the Science

**DOI:** 10.3390/nu5103854

**Published:** 2013-09-26

**Authors:** Deanna L. Pucciarelli

**Affiliations:** Department of Family and Consumer Sciences, Ball State University, Muncie, IN 47304, USA; E-Mail: dpucciarelli@bsu.edu; Tel.: +1-765-285-4791; Fax: +1-765-285-2314

**Keywords:** cocoa, heart-health, nitric oxide, cardiovascular disease, medical history

## Abstract

The medicinal use of cocoa has a long history dating back almost five hundred years when Hernán Cortés’s first experienced the drink in Mesoamerica. Doctors in Europe recommended the beverage to patients in the 1700s, and later American physicians followed suit and prescribed the drink in early America―*ca.* 1800s. This article delineates the historic trajectory of cocoa consumption, the linkage between cocoa’s bioactive-mechanistic properties, paying special attention to nitric oxides role in vasodilation of the arteries, to the current indicators purporting the benefits of cocoa and cardiovascular health.

## 1. Introduction

The link between chocolate and health has been recorded at least as long as Cortés arrival in Mesoamerica [1]. Codices document that chocolate was drank to maintain health in the 1500s. This article will focus on the bioactive mechanistic linkages between cocoa and heart health with special attention given to nitric oxide and its associated history with the Nobel Prize. Interestingly, recently, the prize was linked again with chocolate; this time, however, the author correlated statistics measuring countries’ consumption levels of chocolate and Nobel Laureate winners to argue that chocolate consumption provided a cognitive advantage [2]. Although the early adopters did not have access to current technology to analyze subcellar biomedical benefits, they recognized health-promoting benefits of the era. Researchers continue to investigate the role cocoa plays in heart health, and evidence suggests that there are many positive attributes cocoa provides to the consumer.

## 2. Linkage between Cocoa and Heart Health: Early Beginnings

Atherosclerosis, as a known disease, dates back to at least the time of the pharaohs. Archaeologists have uncovered atherosclerotic plaques in ancient Egyptian mummies [3]. The Greeks, too, referenced heart disease. Associations between coronary heart disease (heart attacks) and weight were noted by Hippocrates in his text, Aphorisms: “Persons who are naturally of a full habit diet suddenly, more frequently than those who are slender” [4].

One of the first nutria-chemical links discovered between heart health and chocolate was indirect and not fully appreciated from original discovery until 150 years later when technology advanced and analytical tools were able to explained one of cocoa’s physiological mechanisms. Ascanio Sobrero (1812–1888) traveled from Turin, Italy, to Paris, in the mid-19th century, to work under the renowned chemist Theophile-Jules Pelouze. In Pelouze’s laboratory Sobrero uncovered the reaction whereby mixing glycerol with nitric and sulfuric acids created an explosion, except if the mixture was cooled during the reaction process. This new compound was labeled: nitroglycerine (NG). The observations of Sobrero were the beginning of journey of coincidence at first and ending with biological evidence later between chocolate and heart health. In February 1847, Sobrero lectured to the Accademia delle Scienze di Torino, and demonstrated to the audience his newly found reaction by detonating a small amount of the compound [5]. Records indicate that Sobrero tasted nitroglycerine and found it sweet, but warned “precaution should be used, for a very minute quantity put upon the tongue produces a violent headache” [6]. Four years later, Alfred Nobel sought tutelage in Pelouze’s laboratory. During high school, Nobel had studied under the Russian chemist Nikolai Zinin, who was also a prior student of Pelouze. Nobel’s family was in the road/tunnel construction business in Sweden. Recognizing the financial potential of such a product, Nobel returned with NG to Stockholm. By 1863, Nobel “realized his first epoch-making invention, the Nobel patent detonator” [5]. Nobel was concerned with world peace, supported the humanities, and of course valued scientific discoveries. He bequeathed his entire estate to a trust designed to award those, who through their hard work and discoveries, might change the world. Thus, the origins of the Nobel Prize can be linked back to nitroglycerine.

Nobel suffered from poor health and intense pain related to angina pectoris. He was advised, coincidently, to take NG for his heart complaint. At the time, it seemed incredulous to Nobel to consume a compound utilized in road construction. Seven weeks before his death he wrote:

*My heart trouble will keep me here in Paris for another few days at least, until my doctors are in complete agreement about my immediate treatment. Isn’t it the irony of fate that I have been prescribed N/G 1(nitroglycerine), to be taken internally! They call it Trinitrin, so as not to scare the chemist and the public*.

[7]

Why did the physicians prescribe NG? Twenty years earlier Benjamin Richardson, a medical doctor and researcher working in London, investigated the physiological effects of amyl nitrite that was administered to a frog. The capillaries in the frog’s foot dilated demonstrating the relationship between NG and vasodilatation [8]. Others worked on the physiology and mechanistic pathways of nitrites over the 19th century. William Murrell, a London physician, prescribed NG to patients and published the positive effects NG provided on relieving chest pain [9,10,11,12]. During this period NG was prepared as a liquid and not easily transported. Murrell wrote to British chemist William Martindale requesting that a solid form of the drug be prepared so that patients could consume the drug, regardless of location, when angina pectoris occurred. Murrell suggested placing the drug (hundredth of a grain) in chocolate [13]. At the turn of the 19th century, consequently, NG and chocolate became linked. The public loved this “drug,” while Murrell regretted his request. He believed the chocolate-coated NG pill would be misused and treated as candy; he tried to retract his original suggestion, but was unsuccessful. Nevertheless, he continued to prescribe NG to his patients [14]. Murrell had no way of knowing that an active ingredient in cocoa (flavonoids) would be investigated for its up-regulation of nitric oxide (a derivative of NG).

**Figure 1:**
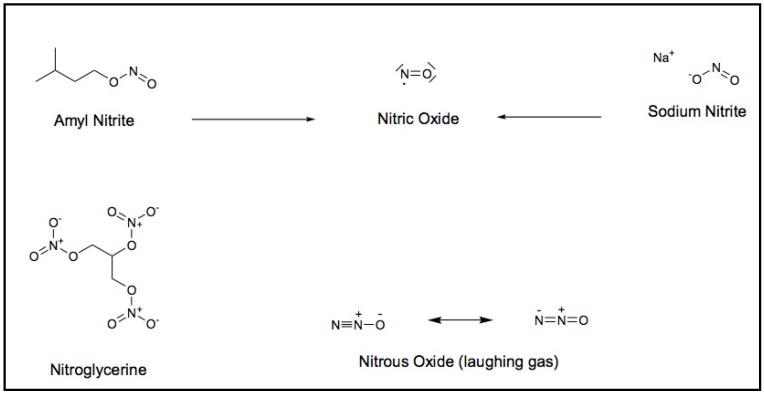
Chemical structures associated with nitric oxide (NO).

A Scotsman and physician, Thomas Lauder Brunton, suggested, as early as 1871, that nitrites produced “vasodilatation due to direct action of the drug on the vessel walls” [15]. The various forms of nitrates (Figure 1) were recognized to produce different physiological effects. The production of Nitric Oxide (NO) from amyl nitrite was determined to be transient, but more substantial than when derived from sodium nitrite. This was due to sodium nitrite’s inability to easily pass the plasma membrane [16]. There was a gap in progress for decades in understanding the physiological mechanisms associated with nitric oxide and the cardiovascular system.

It is not until the 1970s that researchers returned to investigate the relationship between NG and vasodilation of the coronary arteries. A pharmacologist, Fedrid Murad, and colleagues demonstrated that nitrite containing compounds stimulated cyclic guanosine monophosphate (cGMP); cGMP mediates vasodilation.

At the time of this investigation, Murad hypothesized that nitric oxide (NO) was in some way associated with vasodilation, perhaps indirectly through hormones, but lacked evidence to support this hypothesis [17]. Another chemist, Robert Furchgott and his colleague John Zawadzki recognized that soluble guanylate cyclase could be activated by NO, as Murad reported [18]. Gyanylate cyclase plays a role in cGMP production, and thus, is a connection between nitrate compounds and vasodilation. Furchgott and associates proposed that the substance responsible for vaso-relaxation was transient and unstable; they named this substance endothelial derived relaxing factor (EDRF) [19]. Murad and colleagues did not initially identify EDRF as NO, at least not in writing. A third group of pharmacologists, led by Louis Ignarro, published that EDRF was indeed NO [20]. Ignarro’s experiments demonstrated the mechanism whereby guanylate cyclase was activated by NO, and his research findings were published in the December 1987 issue of *Circulation Research* [21]. Another researcher, Salvador Moncada, who like Murad had similar hypotheses, and used similar laboratory techniques drew the same conclusion that EDRF was in fact NO. He published his findings in *Nature’s* 11 June 1987 edition (six months prior to Ignarro’s paper) [22].

On October 12, 1998, the Nobel Assembly at the Karolinska Institute awarded the 1998 Nobel Prize in Physiology or Medicine to Furchgott, Ignarro and Murad for their “discoveries concerning nitric oxide as a signaling molecule in the cardiovascular system” [23]. Moncada’s earlier work was, unfortunately, overlooked. The Nobel Prize founded on the discovery of nitroglycerine 150 years earlier, which was utilized as a detonator to blow through granite and create tunnels was now recognized, in its related NO derivative, for playing a role in opening up biological tunnels (arteries).

The status and subsequent interest the prize lent to the scientific research on the mechanics of NO is evidenced by tabulating citations listed in PubMed for nitric oxide: 560 citations prior to 1990; 4425 citations, 1990–1994; 11,162 citations, 1995–1999 [5]; and 124,343 citations were available on June 5, 2013. The astounding amount of scholarship produced post-1999 revels that nitric oxide was a topic of interest for many researchers. Other physiological mechanisms and pathways relating to NO were reveled through continued research throughout the final years of the 20th century, and still continuing into the 21st century.

What became obvious to researchers were the associations among NO, blood pressure and heart disease. Heart disease by 1910 had become the most common cause of death in the United States, and by mid-century 50% of all deaths was attributed to heart disease [24]. With mortality so high, investigations into causes and risk factors relating to heart disease increased exponentially. Populations with decreased rates of disease were also studied.

## 3. Linkage between Cocoa and Heart Health: Recent Findings

The Kuna Indians live on the San Blas islands off the coast of Panama. A segment of the Kuna population had migrated to Panama City for economic opportunities and other reasons. Researchers recognized that the Island-Kuna had very low incidence of hypertension when aging, whereas the Mainland-Kuna hypertension levels were similar to other urban dwelling people [25]. Hypertension increases with age and is considered a risk factor for cardiovascular disease (CVD). The Island-Kuna had little age-related hypertension and researchers looked at environmental factors, including diet, that might explain the difference. What they discovered was that Island-Kuna, but not Mainland-Kuna, drank five cups of cocoa per day [26]. Moreover, the type of cocoa the Island-Kuna consumed was determined to be *flavonoid-rich* (900 mg/day) and the differences in low prevalence of hypertension were seen greatest in older rather than younger people [27]. Other factors were investigated, such as differences in tobacco use, but were ruled out as contributory [28].

The relationship between consumption of high levels of cocoa and cocoa containing products and low levels of hypertension also were found in another older population located thousands of miles away in Holland. This prospective study focused on a group of older men (Zutphen Elderly Study) and data were collected 15 years post-baseline. A cross-sectional analysis measuring habitual intake of cocoa-containing products was determined to be inversely associated with high blood pressure and prospectively related to cardiovascular mortality [29]. The association between high levels of cocoa consumption and positive heart health outcomes gained international attention in the mid 1990s, and research to determine the nutrients inherent in cocoa that may play a role in this relationship began in earnest during this time.

Flavonoids a subclass of polyphenols are abundant in fruits and vegetables and manifest in nature in many forms. The most commonly consumed and richest source of flavonols in foods are quercetin and kampferol [30]. Although generally low in concentration (~15 to 30 mg/kg) on a wet-weight basis, these nutrients are found in onions, apples, and blueberries, products that are core foods in most American diets [31].

**Figure 2:**
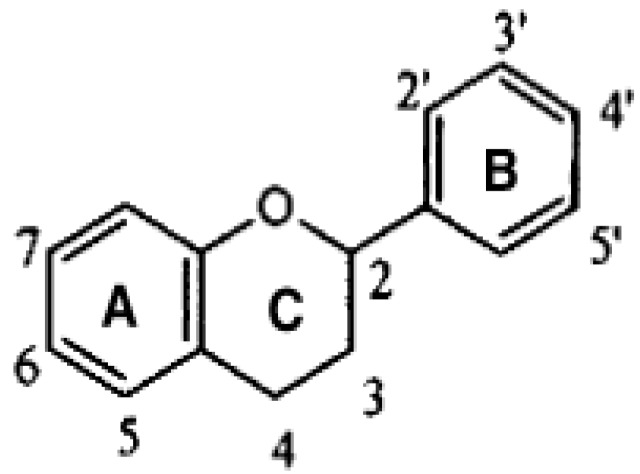
Basic structure of a flavonoid.

Flavonoids have a basic three ring structure (Figure 2) and subclasses are defined by their differing structural properties (moieties). Cocoa and chocolate contain flavan-3-ols, as does tea and wine [32,33]. Flavan-3-ols are subdivided again based on differing moieties with catechin and epicatechin (monomers) and proanthocyanidins (oligomers) comprising the predominate classes found in cocoa. Catechin (+) and epicatechin (−) chemically differ on the 3rd carbon on the *C*-ring (Figure 2). Catechin has a hydroxyl group on the 3rd carbon whereas epicatechin has a hydrogen group. Proanthocyanidins, which make up to 60% of the polyphenol content of fresh cocoa beans has repeating units connecting at the 4th carbon on the *C*-ring [34]. Flavonoids present in the fresh cocoa bean, however, differ from the processed cocoa bean.

Cocoa beans are grown, typically, only within a narrow band defined by 20° north and south of the equator, with the majority of cocoa production harvested in the 21st century in West Africa. Although numerous varieties are known, there are three main botanical cocoa varieties produced for the general market: Criollo (not widely grown), Forastero, and Trinitaro (a crossbreed of the two) [35]. After harvesting pods from the trees, the beans are removed from the white pulpy interior and dried in the sun. Bacteria, naturally intrinsic to the beans, are exposed to wild yeasts floating in the air resulting in microbial fermentation. What is important to the manufacturer is the degree to which fermentation occurs for this processing step impacts final flavor nuances. For those manufacturers interested in flavonoid content, this step is critical as well. End products at this stage (alcohols and organic acids), such as lactic acid, and some researchers suggest d-amino acids are released. These products diffuse into the beans killing the bacteria and produce a specific color and taste aroma [36]. Flavonoid content also is affected by the fermentation process [37].

Roasting the cocoa beans is the next step in cocoa production. Aroma and flavor profiles develop predominantly during the drying and fermentation steps; however, roasting (via Malliard reactions) impacts flavor nuances [38,39]. Processing cocoa beans takes place both on large plantations in addition to thousands of small plots throughout the Ivory Coast, Ghana, and elsewhere. Consequently the end product (cocoa liquor) is subject to wide variability in processing and quality. Maintaining quality control is an issue for most cocoa bean buyers. Not only do flavor profiles change in response to cocoa processing, flavonoid content varies in direct relation to drying, fermentation, and roasting times [40]. Moreover, flavonoids impart astringency to chocolate: with increased flavonoid content on a per gram basis the greater the sensory property of bitterness is perceived [41,42].

Given the interest in *foods for health*, high prevalence of CVD in the United States and increasingly elsewhere, and the reported positive relationship between cocoa and heart health, researchers continue to investigate the varying roles that flavonoids play in human physiology. The research findings are not without controversy, however. In a 2008 review article *Cocoa and Health: A Decade of Research* the authors suggested that stricter controls are needed in clinical trials to determine the correlation between flavon-3-ols and heart health [43]. The authors implied that many of the studies lacked scientific rigor. Nevertheless, a significant body of scholarship indicates cocoa has properties that support positive heart health.

Although diet-disease causality cannot be drawn from epidemiologic studies because of the difficulty of controlling for spurious environmental factors, they are nevertheless worthy of consideration when creating hypotheses on nutrient-disease interactions. Chronic disease typically develops over time and manifests later in older populations. Analyzing cohort food intake may elucidate dietary patterns beneficial to good health. Quantifying exact nutrient consumption is problematical as flavonoids are not easy to measure, measurements are expensive and the composition databases would be very large because of all the different compounds needed to be included [44,45]. Even with the enormity of the task, United States Department of Agriculture (USDA) undertook the challenge and produced a food composition flavonoid database in 2003 [46]. Alerted to overestimation of catechin values in cocoa products, a revised version was issued January 2007, which contains 385 food items [47]. Although this is an impressive beginning, more cocoa product composition values need to be added to the database so that American consumption patterns are better represented.

Standardizing the amount of catchins/g cocoa in different manufactured cocoa products has been discussed; however, this variant must be considered when correlations between “natural” cocoa intake and positive heart health are claimed. To control for the variation of catechins in natural cocoa, scientists working in research laboratories utilize synthetic cocoa products designed to contain a set amount of flavon-3-ols to ensure reproducible results. Thus, what takes place in the laboratory may not reflect cocoa consumption in the free market [48]. Further, in interpreting study results, it is important to realize that the manufactured intervention cocoa may not represent consumer product availability.

Another consideration in determining evidence that flavanoids are heart health beneficial is the degree of nutrient bioavailability at the post-absorption level [49,50]. There are nearly 100 reports on the bioavailability of flavonoids in humans and almost that number in clinical trials on bioactivity [48]. What is tested in the laboratory is non-native (e.g., manufactured) flavanoids. It has been widely reported that there are major differences in bioavailability between the native and non-native compounds [51,52]. To complicate the analyses further, subsets of literature that focused on the bioavailability of flavon-3-ols (which had been methylated or galloylation) were not consistently considered [48,53]. Given that these processes are determinants to absorption and thus bioavailability, comparing results across papers becomes corrupted [31,54,55]. In spite of these limitations researchers continue to measure the relationship between cocoa consumption and oxidative stress. A considerable body of literature indicates that oxidative stress is involved in the pathogenesis of chronic diseases and a daily contribution of flavanoids may reduce that stress load [56,57,58].

Mechanistic research that focused on the relationship between flavonoids and cardiovascular and/or coronary heart disease (Heart Health) beginning mid-1990s and continuing to present day can be clustered under four headings: (1) anti-oxidant properties; (2) vasodilation of smooth muscle; (3) anti-platelet aggregation; and (4) anti-inflammatory properties. This review collates and summarizes data from human clinical trials, epidemiologic studies and animal experimental investigations and identifies salient findings in each of the four pharmacokinetic areas.

One method to measure the relationship of antioxidant capacity *in vivo* is to measure a biomarker for redox regulation at the transcriptional level. Jan Moskaug, and co-workers used a mouse model and reported that flavonoids modulated expression of the rate-limiting enzyme γ-glutamylcysteine synthetase, a significant finding since this enzyme regulates glutathione, and Moskaug and co-workers asserted that glutathione is the most important endogenous antioxidant in cells [59]. Others have suggested that even if the level of bioavailability of post-absorptive flavonoids is lower than previously reported, localized redox reactions in the gastrointestinal tract may be significant [60]. Suggested effects include binding of pro-oxidant iron and inhibition of cyclooxgenases and lipooxgenases, destructive agents of cellular membranes [61]. Adverse effects of high flavonoid consumption can occur as well. Flavonoids have a high affinity for chelating iron and in individuals with marginal iron status high flavonoid intake can increase risk of iron depletion [42,62]. The scientific community lacks consensus regarding the measure of antioxidant effect provided in the diet from cocoa flavonoid consumption [63,64,65]. In clinical, epidemiological and experimental literature contradictory results appear regarding the association between increased flavonoid intake and antioxidant capacity while researchers acknowledge that populations who consume increased levels of flavanoids have a lower incidence of myocardial infarction [66,67]. Researchers conclude that more clinical-control, intervention studies are needed to confirm that cocoa, at the biological-available level, exerts antioxidant protective effects.

Even before the 1998 Nobel Prize in Physiology or Medicine was granted to Furchgott, Ignarro and Murad for their discoveries concerning nitric oxide as a signaling molecule in the cardiovascular system [68], the relationship between cocoa and NO expression was beginning to be heavily investigated. It was generally accepted that increases in NO production vasodilates smooth muscle, such as aortic rings [53]. Heart disease and CVD patients lack vascular homeostasis. One function of the endothelium is to maintain normal vascular tone by balancing the production of vasodilators and vasoconstrictors [69]. It was suggested that by daily consuming flavonoid foodstuffs positive vasodilator factors would counterbalance negative effects that hinder vascular tone.

The next logical (and profitable) step for chocolate/cocoa manufacturers to take was to demonstrate that flavonoids, inherent in cocoa, up-regulated NO production. In the mid-1990s clinical trials began in earnest to examine the dose-response between cocoa consumption and up-regulation of NO activity resulting in improved vascular tone [70].

It had been known that populations that consume high intakes of plant-derived foods and beverages were inversely associated with risk of CVD, and one of the beneficial effects of the plant-based diet had been frequently ascribed to flavanols, a subgroup of flavonoids present in fruits, vegetables, and cocoa specifically.

Studies in human trials suggested that vascular effects of flavanols are due, in part, to increased nitric oxide synthase levels consequently up-regulating bioactive NO activity [71,72,73]. The consequence of increased NO activity is an increase in artery dilation. In a randomized, double-blind, crossover study involving 11 participants (self-described smokers), 100 mL cocoa drink with either high (176 to 185) or low (<11 mg) flavonol content was consumed on two separate days. Flow mediated dilation (FMD) increased in the high flavanol group (4.5% ± 0.8% to 6.9% ± 0.9%, *p* < 0.05) 2 h post ingestion [74].

In another clinical trial with eight healthy male adults, Hagen Schroeter and co-workers demonstrated that (a) pure epicatechin quantitatively mimics the vascular effects of flavanol-rich cocoa; and, (b) inhibition of nitric oxide synthase (NOS) in humans and aortic rings “abolishes vascular effects” as measured by FMD [75]. These findings suggest that, indeed, cocoa improves vascular tone. Schroeter’s results were corroborated by another small clinical study that included six male smokers. The investigators measured the response in FMD after consuming 306 mg of flavanols (procyanidins, epicatechin, catechin) three times daily for seven days. The study participants were asked to refrain from smoking and required to fast 12 h prior to study visits so that confounding results could be minimized. Sustained increase in FMD of the brachial artery increased with repetitive consumption of the high-flavanol cocoa drink, though, the effect was temporal [76]. Combined, these studies suggest a positive effect on FMD when participants consumed high levels of cocoa-derived flavanols. However, a group of Australian researchers found different results.

In a six-week randomized, double-blind placebo controlled study, forty subjects diagnosed with coronary artery disease consumed either a flavanol-rich chocolate bar (444 mg/day) and cocoa beverage daily or matching isocaloric placebos (19.6 mg/day) Brachial artery FMD was measured at baseline, 90 min following the cocoa intake and after three and six weeks of daily consumption. Between baseline and six weeks no acute or chronic changes in FMD were seen in either group [77]. Another group of researchers found that with 32 postmenopausal hypercholesterolemic women randomly assigned to consume a high flavanol beverage (446 mg/day) or low flavanol beverage (43 mg/day) for six weeks, (essentially mimicking the Australian study) brachial artery hyperemic blood flow increased by 76% (*p* < 0.05) [78]. Clearly, more clinical-controlled crossover double-blind studies are needed to confirm strong clinical endpoint evidence.

Studies indicate aspirin vasodilates arteries as well [79], however, aspirin is more commonly associated with reducing platelet aggregation, a CVD risk factor. Platelet activation in damaged arteries can lead to thrombus formation. Platelets are activated after adhesion to collagen that becomes exposed and this leads to release and synthesis of agents such as adenosine diphosphate (ADP) and thromboxane A_2_ (TXA_2_), which induce platelet aggregation. As the platelets build up with fibrin an occlusion can occur causing a stroke, heart attack, or acute angina. Activated platelets can interact with leukocytes via P-selectin leading to further inflammation [80].

It has been suggested that moderate to high cocoa intake may also reduce platelet adhesion via effects of flavanoids on signal transduction pathways [81]. Cocoa (897 mg epicatechin and oligomeric procyanidins combined) has been shown to reduce ADP and adrenaline-induced expression of the activated platelet glycoprotein (GPIIa/IIb), a regulator in platelet aggregation [82,83]. The former study, although short term, has been replicated in a longer four-week study whereby subjects consumed moderate amounts of cocoa flavanols. Endpoints measured were P-selectin protein measured by platelet cells, which decreased, in addition ADP-induced platelet aggregation and platelet volume declined as well [84]. Flavonoids may exhibit a positive reciprocal relationship between NO-related mechanisms and platelet function creating a dual health promoting environment.

Pathogenesis of chronic and acute inflammation leading to CVD is multifactorial, but it is generally accepted that initiation and promotion are associated with cytokines and eicosanoids [85,86]. Some of the significantly studied pro-inflammatory types include: interferon-γ, interleukin (IL)-1α, and IL-6 [87]. Additionally a few of the enzyme systems involved in the inflammatory response include xanthine oxidase and NADH/NADPHA oxidase groups [88]. Flavanol-rich beverages have been shown to increase NO production and reduce xanthine oxidase [89,90]. As with other cocoa research, many of the investigations are predominately *in vitro* experiments and results may not be expressed in humans.

Further, studies of *in vitro* stimulation measured the response to peripheral blood mononuclear cells (PBMCs) isolated from healthy volunteers and cultured with cocoa flavanol fractions that differed by chain length (monomers and procyannidin dimmers that could be found in human circulation after consuming cocoa flavanols) [91]. The chain length had an effect on cytokine release from both unstimulated and stimulated PBMCs, and the researchers concluded that oligomers were potent stimulators of the immune system and supported anti-inflammatory cytokines.

A group of researchers investigated LDL oxidation because it was associated with the atherosclerotic process. Michael Peluso has suggested that flavonoids may suppress LDL oxidation and inflammatory progression in the artery wall and researchers working with animal (rabbit) models support his theory [92]. In a comparative, double-blind study, researchers examined 160 subjects who ingested either cocoa powder containing low-polyphenolic compounds (placebo-cocoa group) or three levels of cocoa powder containing high-polyphenolic compounds (13, 19.5, and 26 g/day for low-, middle-, and high-cocoa groups, respectively) for four weeks. On two occasions per day, the prepared beverages were consumed. Blood samples were collected at baseline and then at week four after intake of the test beverages. Plasma oxidized LDL concentrations decreased in all groups compared with baseline. A stratified analysis was performed on 131 subjects who had a LDL cholesterol concentrations of > or = 3.23 mmol/L at baseline. In these subjects, plasma LDL cholesterol, oxidized LDL, and apo B concentrations decreased, and the plasma HDL cholesterol concentration increased, relative to baseline in all groups. The results suggest that polyphenolic substances derived from cocoa powder may contribute to a reduction in LDL cholesterol, and an elevation in HDL cholesterol [93], a positive change in markers for CVD risk.

Utilizing a rabbit model, researchers measured the plasma concentration of thiobarbituric acid reactive substances (TBARS), which is a lipid-peroxidation index. Researchers reported a significant TBARS decrease one month after the start of cocoa liquor polyphenols [CLP] administration compared to that of the control group. An antioxidative effect of CLP on LDL was observed from two to four months of administration [94].

Other researchers have suggested a new use for cocoa bean skins as good sources of insoluble fiber—a product known for its cholesterol-lowering properties, however, bitter sensory properties would need to be overcome [95]. While some of these studies report conflicting results, many more suggest that flavonoids play positive roles in immune integrity. Other studies focusing on cocoa positive health outcomes include: increase in insulin sensitivity and decrease in blood pressure [96,97,98], cocoa as gastro-protective [99] cocoa and improved cognition [100], cocoa blocks UV-induced erthema and improves skin condition [101], and cocoa inhibits growth of breast cancer cells [102].

As Cesar Fraga has pointed out in an editorial review, interpretations of results are uncertain because of the lack of rigor in documenting the experiments details, such as: amount of polyphenols may be listed, but how they *quantified* the dosage often is left unreported [103]. It seems that cocoa has become a panacea for today’s many chronic diseases, and that we have returned to the 17th century where, prior to laboratory analyses and precise instrumentation, physicians claimed that cocoa was indeed the food of the gods and the healer of all aliments [104].

## 4. Concluding Comments on the Relationship between Cocoa and Heart Health

Epidemiologic and experimental studies continually suggest a positive relationship between polyphenols consumption natural in cocoa and heart health [105,106]. The question that remains is whether these data are relevant for human disease outcomes where polyphenols are typically consumed at low concentrations (in the United States) [32]. What is compelling, however, is that researchers globally who studied cocoa flavanols mechanistic pathways have agreed that cocoa flavonoids—*in vitro* and through animal models—upregulate enzymes that act as a vasodilator of the coronary arteries. European physicians, too, regularly have cited American investigations on cocoa and endothelial function [107]. The European Food Safety Authority, in response to Barry Callebaut application for a scientific substantiation of health claims related to cacao flavanols and maintenance of normal endothelium-dependent vasodilation, issued the following opinion, “cocoa flavanols help maintain endothelium-dependent vasodilation, which contributes to normal blood flow”. In order to obtain the claimed effect, 200 mg of cocoa flavanols should be consumed daily [108]. The linkage between cacao consumption and heart health goes back many years, has been and continues to be a popular area of scientific interest. With increased positive outcomes in human studies that include 200 mg of cocoa flavanols the public will benefit from the scientific evidence.
